# Synergistic immunomodulation: integrating physical ablation with immune checkpoint inhibitors for metastatic colorectal carcinoma

**DOI:** 10.3389/fimmu.2026.1859114

**Published:** 2026-06-02

**Authors:** Xuebing Shi, Changping Wu, Wenxia Deng

**Affiliations:** 1 Cancer Treatment Center, Tongling People’s Hospital, Tongling, Anhui, China; 2 Tongling Academy of Medical Sciences, Tongling, Anhui, China; 3 Department of Tumor Biological Treatment, The Third Affiliated Hospital of Soochow University, Changzhou, Jiangsu, China; 4 Department of Medical Oncology, The Third Affiliated Hospital of Soochow University, Changzhou, Jiangsu, China

**Keywords:** colorectal carcinoma, immune checkpoint inhibitors, immunotherapy, metastases, physical ablation

## Abstract

Colorectal carcinoma (CRC) is one of the most common malignant tumors worldwide, with treatment options including surgery, chemotherapy, radiotherapy, and targeted therapy. However, the overall efficacy of treatment for metastatic CRC (mCRC) remains unsatisfactory. In recent years, immune checkpoint inhibitors (ICIs) have shown good therapeutic effects in some patients, but the overall response rate remains low. Physical ablation (PA) is a localized treatment modality that, in addition to eradicating targeted lesions, promotes anti-tumor immunity by releasing tumor-associated antigens from damaged tumor cells. ICIs enhance this PA-elicited anti-tumor immune response by inhibiting immunosuppressive pathways and unleashing T-cell activity, resulting in a synergistic anti-tumor effect. This article reviews the latest research on the treatment of mCRC with PA (mainly including radiofrequency ablation, microwave ablation, and cryoablation) combined with ICIs, focusing on how PA combined with ICIs can reshape the tumor immune microenvironment to produce a synergistic anti-tumor immune response. Finally, this article also discusses the challenges currently faced by the combined treatment and looks forward to future research directions, aiming to provide a new treatment strategy to improve the clinical prognosis of mCRC.

## 1 Introduction

Colorectal carcinoma (CRC) is one of the most common malignant tumors in humans. In 2022, CRC accounted for 9.6% of newly diagnosed malignancies and 9.3% of cancer-related deaths globally (1). The incidence and mortality rates of CRC ranked third and second, respectively, among all malignant tumors (1). In China, CRC currently ranks second in incidence and fourth in mortality among all malignancies, with both incidence and mortality rates exhibiting a gradually increasing trend (2).

Approximately 25% of CRC patients are already in the advanced stage at the time of initial diagnosis, with an additional 35% experiencing tumor metastasis during progression of the disease (3). Metastatic CRC (mCRC) is most commonly associated with liver metastases (LM), with about 50% of patients experiencing LM during the natural history of CRC (4). Among secondary liver cancers, CRCLM is also typically the most common (5). The inherent characteristics of mCRC (especially CRCLM), such as strong tumor heterogeneity, frequent genetic mutations, immunosuppressive microenvironment, and propensity for drug resistance, significantly complicate traditional treatment approaches. Currently, fully exploring the potential of existing treatments, investigating potential synergistic effects between different therapeutic approaches, and establishing optimal combined treatment strategies are of significant importance for improving the survival rate and quality of life for mCRC patients.

Physical ablation (PA) is a minimally invasive or non-invasive cancer treatment method that destroys and eliminates tumor tissues using physical energy under image guidance, mainly including radiofrequency ablation (RFA), microwave ablation (MWA), photothermal ablation (PTA), high-intensity focused ultrasound ablation (HIFUA), irreversible electroporation (IRE), and cryoablation (CA), among others (6–11). Its application in cancer treatment has become increasingly widespread in recent years (6–11). However, PA also has limitations, including tumor size restrictions, difficulty in controlling the ablation zone, short duration of anti-tumor immune responses, and a high risk of tumor recurrence and progression. Immune checkpoint inhibitor (ICI) therapy is a revolutionary anticancer immunotherapy that overcomes tumor immune escape by blocking inhibitory signals within the tumor microenvironment (TME), leading to the reactivation of endogenous immune cells, particularly T cells, enabling them to effectively attack and clear tumor cells (12). ICI therapy has the advantages of long-lasting efficacy and fewer side effects (12). At present, several ICIs including those targeting cytotoxic T lymphocyte-associated antigen 4 (CTLA-4), programmed cell death protein 1 (PD-1) and its ligand PD-L1 have been clinically available and used either alone or in combination for the treatment of malignant tumors (13–18).

However, not all patients can benefit from ICI therapy. Some patients not only do not respond to the treatment but may even experience disease progression. Additionally, the issue of resistance to ICIs is difficult to overcome (19–21). In recent years, a growing number of studies have shown that combination of PA and ICIs can produce synergistic anti-tumor effects, opening up a promising new direction for the combined treatment of cancers (22–26). The present review offers a comprehensive overview of current research progress concerning the therapeutic strategy of combining PA with ICIs in the treatment of mCRC.

## 2 Literature research

A comprehensive search of the PubMed and Google Scholar databases was conducted to identify original studies on the combination of PA and ICIs for treating mCRC. The search used the following terms: (((“thermal ablation”) OR (“radiofrequency ablation”) OR (“microwave ablation”) OR (“photothermal ablation”) OR (“high-intensity focused ultrasound ablation”) OR (“laser ablation”) OR (“irreversible electroporation”) OR (“cryoablation”)) AND ((“colorectal carcinoma”) OR (“colorectal cancer”) OR (“colorectal tumor”) OR (“MC38 tumor”) OR (“CT26 tumor”)) AND ((“immune checkpoint inhibitor”) OR (“immune checkpoint blockade”) OR (“immunotherapy”))). The search was not limited by date and continued until December, 2025.

## 3 Inclusion and exclusion criteria

Included studies were: (a) Preclinical or clinical studies on the combination of local PA with immune checkpoint blockade in treating mCRC, and (b) those clinical studies that reported follow-up results regarding efficacy and safety. Excluded studies were: (a) Preclinical or clinical studies investigating PA or immunotherapy alone for the treatment of mCRC. (b) Studies on the combination of PA with non-immune checkpoint blockade for the treatment of mCRC.

## 4 PA combined with ICIs

### 4.1 Overview of different PA modalities

RFA utilizes high-frequency electromagnetic waves to generate heat, which directly kills tumor cells and induces inflammatory responses in the TME (27–30). MWA is a minimally invasive treatment method that utilizes microwave energy to generate heat, causing denaturation and coagulation of tumor cell proteins, leading to necrosis of the tumor cells, and thereby eliminating the tumor (31–34). PTA is a highly selective and minimally invasive oncological treatment method based on the principle of converting light energy into localized thermal energy, which precisely destroys the targeted tumor lesions (35, 36). In the great majority of cases, PTA utilizes photothermal agents to generate sufficient heat mainly under near-infrared laser light irradiation to kill tumor cells and induce anti-tumor immunity (35–37). Thermal HIFUA is a completely noninvasive anti-tumor treatment modality, which converges external ultrasonic waves at the focus to produce instantaneous high temperature and ablate the targeted tumor (38–42). RFA, MWA, PTA and HIFUA which are all thermal ablation techniques cause coagulative necrosis of tumor cells in treating cancers (27, 31, 35, 38).

IRE is an emerging nonthermal ablation technology that primarily relies on the nonthermal effects generated by electric fields to destroy cells (43). The cell death induced by IRE is mainly apoptosis (43). Currently, IRE has been applied in the treatment of various malignant tumors (6, 44–47). CA is a minimally invasive tumor treatment method that rapidly cools tissues to form ice crystals, which disrupts cellular membranes, blood vessels and other structures (48). This process leads to cell dehydration, protein denaturation, and ultimately induces tumor cell necrosis (48). CA has exhibited some therapeutic effects in various malignant tumors (49–54).

All of above PA modalities not only can eliminate targeted tumors, but also release tumor-associated antigens (TAAs) and damage-associated molecular patterns (DAMPs), which enhances the body’s anti-tumor immune response, reshapes the tumor immune microenvironment, induces immunogenic cell death and exhibits abscopal effects on nonablated tumors (27, 28, 33, 35, 39, 43, 48, 55). However, in the majority of cases, PA alone is unable to produce sufficiently robust anti-tumor immune responses to completely eliminate residual tumors and prevent recurrence (29, 30, 43, 47). ICIs can relieve tumor-induced suppression of the immune system and improve the killing capacity of immune cells, particularly CD8^+^ T cells, and the combination of PA with ICIs exhibits a synergistic anti-tumor effect (34, 40, 42, 56–59).

### 4.2 Upregulation of immune checkpoint expression following PA in murine mCRC models

In murine models of mCRC, PA has been demonstrated to upregulate the expression of multiple immune checkpoints within the TME of nonablated lesions. Shi et al. (60) found that RFA could elevate the expression of both PD-1 and PD-L1 in the untreated tumor. In two separate studies, Chen et al. (61) and Shao et al. (62) observed that after ablating one tumor using MWA, there was a significant upregulation of T-cell immunoglobulin and ITIM domain protein (TIGIT) expression and lymphocyte-activation gene 3 (LAG-3) expression on CD8^+^ T cells in the contralateral lesion. Furthermore, it was also demonstrated that MWA increased the expression of PD-1 on tumor-infiltrating T cells and the expression of PD-L1 on tumor cells within the nonablated lesions (63, 64).

Therefore, PA can promote the expression of immune checkpoints in the TME, thereby enhancing tumor sensitivity to ICI therapy. This provides a crucial theoretical basis for the combination therapy of PA with ICIs for mCRC.

### 4.3 PA in combination with a single ICI

In multiple preclinical studies based on murine mCRC models, PA, including RFA (60), MWA (63), PTA (65, 66) and IRE (67), combined with PD-1 blockade therapy exhibited synergistic anti-tumor effects. The combination treatment significantly inhibited the growth of unablated tumors and prolonged the overall survival (OS) of mice. Mechanistic studies demonstrated that PA in combination with anti-PD-1 antibody increased the proportion of CD8^+^ T cells (63, 67), activated the CXC motif chemokine ligand 10 (CXCL10)/CXC motif chemokine receptor 3 (CXCR3) signaling pathway (63), and enhanced the anti-tumor immunity of T cells within the TME of unablated lesions (60).

Other *in-vivo* studies using murine bilateral MC38 or CT26 tumor models have indicated that the combination of PA (MWA or PTA) with PD-L1 blockade yields synergistic anti-tumor efficacy (64, 66, 68). In the combination treatment group, the growth of nonablated tumors was markedly suppressed, and the mice exhibited significantly prolonged survival times (64, 68). Mechanistic investigations revealed that the combination therapy activated antigen-presenting cells, reversed the immunosuppressive TME, induced immunogenic cell death, promoted the interferon-γ (IFN-γ)/CXCL9/CD8^+^ T cell-mediated anti-tumor immune response (64, 66, 68).

TIGIT is an emerging immune checkpoint expressed primarily on effector/regulatory CD4^+^ T cells, follicular helper CD4^+^ T cells, effector CD8^+^ T cells, and natural killer (NK) cells (69). Recently, Chen et al. (61) evaluated the therapeutic potential of combining MWA with anti-TIGIT antibody in a murine bilateral MC38 tumor model. The combination therapy significantly suppressed tumor growth and prolonged survival compared to either modality alone. Mechanistically, flow cytometry revealed a heightened infiltration of total CD8^+^ T cells, IFN-γ^+^CD8^+^ T cells and tumor necrosis factor-α (TNF-α) positive CD8^+^ T cells in the TME. Single-cell transcriptomic analysis further demonstrated that MWA combined with TIGIT blockade significantly upregulated CXCR3 expression in CD8^+^ T cells, facilitating their tumor infiltration. Furthermore, the combination therapy reshaped the TME by weakening myeloid-mediated suppression while enhancing T-cell crosstalk. The CC motif chemokine ligand (CCL)/CXCL signals in CD8^+^ T cells of the combination therapy group were also significantly increased. These findings suggest that the synergy between MWA and anti-TIGIT antibody is mediated by the relief of TIGIT-driven inhibitory signals and the promotion of CCL/CXCL-directed T-cell recruitment.

LAG-3 is an inhibitory receptor prevalent on T cells, NK cells, plasmacytoid dendritic cells (DCs), and Treg cells, playing a significant role in driving T-cell exhaustion (69). In a preclinical study, Shao et al. (62) demonstrated that combining MWA with anti-LAG-3 therapy yielded superior anti-tumor efficacy, significantly slowing tumor growth and extending survival compared to monotherapy. Flow cytometry analysis revealed an increased infiltration of CD4^+^ T and CD8^+^ T cells, with a higher proportion of effector CD8^+^ T cells (IFN-γ^+^ and TNF-α^+^) within the TME. Furthermore, single-cell transcriptomic profiling confirmed the upregulation of *Ifng* and *Tnf* genes in CD8^+^ T cells. The combination therapy also reshaped the TME by enhancing myeloid-to-CD8^+^ T cell crosstalk and enriching IFN and CXCL signaling pathways. These results underscore the potential of MWA combined with LAG-3 blockade to reverse immune exhaustion and promote a more immunostimulatory microenvironment.

Collectively, these preclinical investigations strongly suggest that combining PA with a single ICI can significantly potentiate anti-tumor immune responses, leading to synergistic anti-tumor efficacy. This combination therapy holds considerable promise as a potential treatment strategy for mCRC and warrants further rigorous clinical investigation and exploration.

### 4.4 PA in combination with dual ICIs or with a single ICI plus an immunomodulator

Wehrenberg et al. (59) established bilateral MC38 tumor models in mice to evaluate the anti-tumor effect of CA combined with dual ICIs. In the combination treatment group, mice received intraperitoneal injections of anti-PD-1 antibody and anti-CTLA-4 antibody, followed by CA treatment administered to one of the tumors. The OS of mice in the combination therapy group was superior to the control and monotherapy groups. Further research indicated that CA increased the level of activated CD8^+^ T cells and reduced the level of exhausted CD8^+^T cells in contralateral nonablated tumors. Moreover, CA induced an increase in various cytokines and chemokines, which played pivotal roles in the TME, contributing to elevate the anti-tumor immunity and correlating with improved tumor response to dual ICIs therapy. In an *in-vivo* study, Han et al. (70) observed that combining HIFUA with the immune adjuvant (PLGA-R837) plus anti-CTLA-4 antibody not only significantly inhibited the growth of distant, nonablated tumors but also markedly prolonged the survival time of tumor-bearing mice. Mechanistic analysis indicated that this combination therapy led to increased CD8^+^ T cell infiltration and decreased regulatory T (Treg) cell populations in the distant tumor tissues. Furthermore, the levels of IFN-γ and TNF-α were significantly elevated.

Wu et al. (34) found that MWA combined with anti-PD-1 antibody and interleukin-21 exhibited enhanced distant anti-tumor immune responses in a murine bilateral MC38 tumor model. In other murine mCRC models, investigators have demonstrated that combining RFA with a single ICI plus either immune adjuvants or tumor vaccines can significantly improve adaptive anti-tumor immune responses by increasing T-cell infiltration and enhancing the effector function of CD8^+^ T cells within the TME of nonablated tumors (70–72).

All in all, above preclinical studies have indicated that PA combined with dual ICIs or with a single ICI and an immunomodulator can markedly boost anti-tumor immunity and ameliorate the immunosuppressive TME, providing a potential therapy for mCRC, but further clinical research is still required for validation.

### 4.5 Incomplete PA in combination with ICIs

By using mouse CT26 and MC38 tumor models, Shi et al. (73) verified that incomplete RFA (iRFA) could promote tumor development and hinder the efficacy of PD-1 inhibitor. A further mechanistic study showed that in the local inflammatory environment induced by iRFA, the C-C motif chemokine ligand 2 (CCL2) produced by tumor cells promoted continuous accumulation of myeloid-derived suppressor cells (MDSCs), which in turn suppressed the function of T cells in the TME. Blocking the CCL2/C-C motif chemokine receptor 2 (CCR2) signaling axis could enhance the anti-tumor activity of anti-PD-1 antibody, providing a potential salvage therapy for residual tumors after iRFA.

Ren et al. (74) constructed a murine model of CRCLM by injecting CT26 cells intrasplenically. After treating the LM with iRFA in each group of mice, they administered intraperitoneal injections of melatonin and/or PD-L1 inhibitor. The combination of melatonin and anti-PD-L1 antibody had the best inhibitory effect on residual tumors, significantly superior to monotherapy with melatonin or PD-L1 inhibitor. Further studies revealed that melatonin could improve the hypoxic state and immunosuppressive microenvironment of residual tumors after iRFA, increase the infiltration of CD8^+^ T cells in the TME, reduce the proportion of MDSCs, and enhance the efficacy of anti-PD-L1 antibody, thereby inhibiting tumor progression, which provides another salvage treatment option for residual tumors following iRFA.

In summary, whereas complete RFA establishes an immune-stimulating TME, iRFA conversely fosters an immunosuppressive TME. This highlights a critical clinical caution regarding ablation margin control. In clinical practice, efforts should be made to select appropriate target lesions, ablation margins, ablation parameters, and ablation duration for complete ablation, thereby avoiding incomplete ablation. Should incomplete ablation occur, blocking the CCL2/CCR2/MDSCs pathway with CCR2 antagonists or administering melatonin therapy can be considered as salvage treatment strategies.

## 5 Rationale analysis for the synergistic anti-tumor effects of PA combined with ICIs

**Figure 1:**
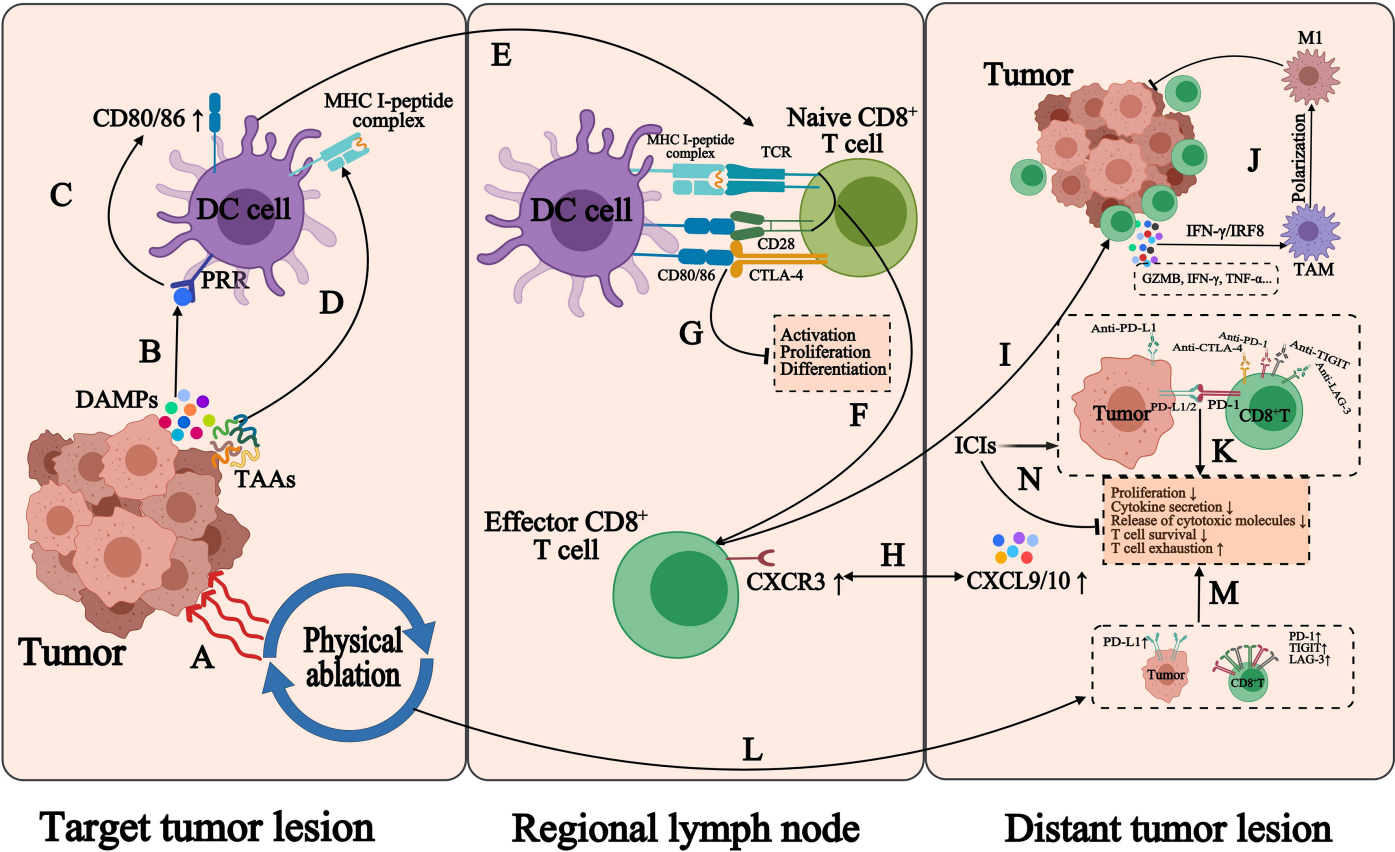
Anti-tumor immune response mediated by PA combined with ICIs. **(A)** PA destroys the target tumor lesion. After ablation, the target lesion undergoes necrosis and releases TAAs and DAMPs. **(B)** DAMPs bind to pattern recognition receptors (PRRs) on the surface of APCs mainly including DCs. **(C)** The co-stimulatory molecules CD80/86 on DC surface are up-regulated. **(D)** The TAAs are processed by DCs and digested to short peptides, subsequently antigenic peptides bind to the major histocompatibility complex-I (MHC-I) on the surface of DCs, forming MHC-I-peptide complexes (MPCs). **(E)** DCs migrate to regional lymph nodes, and interact with naive CD8^+^ T cells. **(F)** MPCs bind to T cell receptors (TCRs) on the surface of naive CD8^+^ T cells. Additionally, the co-stimulatory molecule CD28 on naive CD8^+^ T cells binds to CD80/86 on DCs. Naive CD8^+^ T cells are activated, then proliferate and differentiate into effector CD8^+^ T cells. **(G)** The co-inhibitory molecule CTLA-4 on the surface of CD8^+^ T cells competitively binds to CD80/86 on the surface of DCs, thereby inhibiting the activation, proliferation, and differentiation of CD8^+^ T cells. **(H)** The expression of CXCR3 on the surface of effector CD8^+^ T cells is increased, and the levels of CXCL9/10 in the distant nonablated TME are elevated. **(I)** Effector CD8^+^ T cells migrate to distant tumor tissues via the CXCL9/10/CXCR3 signaling axis and release effector molecules, including IFN-γ, TNF-α, and GZMB, exerting cytotoxic effects on tumors. **(J)** IFN-γ promotes the polarization of TAMs into M1 macrophages via the IFN-γ/IRF8 signaling axis, which exerts tumor- suppressive effects. **(K)** The PD-1 of CD8^+^ T cell binds to PD-L1/2 on the surface of tumor cell, inhibiting the proliferation of CD8^+^ T cells, reducing the secretion of cytokines and the release of cytotoxic molecules, and promoting the exhaustion of CD8^+^ T cells. **(L)** PA promotes the expression of immune checkpoints in nonablated distant tumor lesions. **(M)** The increased expression of immune checkpoints inhibits the proliferation of CD8^+^ T cells, reduces the secretion of cytokines and cytotoxic molecules, and promotes the exhaustion of T cells. **(N)** The administration of ICIs relieves the immunosuppression of T cells in the TME. This figure was created with MedPeer (medpeer.cn).

As a local treatment modality, PA combined with ICIs has the potential to synergistically enhance anti-tumor effects. PA exerts its anti-tumor effects through multiple mechanisms, including direct killing of tumor cells, releasing TAAs and DAMPs, altering the local immune microenvironment, and exhibiting abscopal effects (27, 28, 31, 32, 35, 38, 43, 48, 55). ICIs can release T cell immune suppression, enhance the cytotoxicity of T cells, and promote the formation of memory T cells, producing durable anti-tumor effects (12, 75, 76). When these two modalities are combined, the TAAs and DAMPs released from the targeted tumor by PA can enhance the immune system’s recognition of the tumor, promoting the activation, proliferation, effector differentiation of CD8^+^ T cells and migration to the nonablated tumor sites. Simultaneously, ICIs can increase the immune activity of CD8^+^ T cells, thereby enhancing the killing effect on tumor cells. PA, as a local treatment, can reduce tumor burden and control local tumors, while ICIs, as a systemic therapy, can control distant lesions, prevent tumor recurrence and provide long-term protection. The combination of PA and ICIs highlights the complementary nature of local and systemic treatments, working together to achieve a better anti-tumor efficacy (77, 78). **Figure 1** illustrates the anti-tumor immune response mediated by PA combined with ICIs.

## 6 The characteristics of different PA modalities and the selection of ablation methods and sites in clinical practice

In recent years, PA has been increasingly widely applied in the clinical treatment of localized tumors. Each PA method possesses its own advantages and disadvantages. **Table 1** summarizes the characteristics of different PA methods.

**Table 1:** Characteristics of different PA methods.

Ablation method	Main source of energy	Mechanism of action	Advantages	Disadvantages	Clinical indications	Contraindications	Reference
RFA	High-frequency electrical current	Heat energy produced by high-frequency electric current, coagulation necrosis	Established safety profile and wide availability, mature technology and simple operation	High rates of incomplete ablation for tumor diameters of 3-5 cm, thermal sink effect, high risk of damage to surrounding vital structures	Applicable to small size (usually diameter< 3-4 cm), a limited number of solid tumors, such as early hepatocellular carcinoma, liver metastases (especially of colorectal origin), early stage or inoperable lung carcinoma, early localized renal cell carcinoma, pain control of bone metastases, thyroid nodule	Proximity to vital organs/vessels, severe coagulopathy, inability to localize/immobilize tumor	(79–81)
MWA	Microwave	Water molecules vibration caused by microwave, rapidly producing heat, coagulation necrosis	Rapid heating, larger ablation range (potential treatment of tumor diameters ≥ 3 cm), less affected by the thermal sink effect	Rapid energy attenuation, poor penetration	Suitable for large volume (up to 5-7 cm) or rich blood supply tumors, including larger primary or metastatic liver lesions, lung carcinoma of moderate size, early localized renal cell carcinoma	Proximity to vital structures, severe coagulopathy, metal implants near tumor	(81–83)
PTA	Near-infrared laser light	Laser energy converted into heat energy	Good targeting, selective destruction of tumor cells, less damage to surrounding tissues, shorter ablation time	Requiring precise laser systems and special photothermal agents, light conduction is affected by tissue, limited effect on deep tumors	Suitable for tumors of limited depth, but requiring high precision ablation, such as tumors of the digestive tract, some prostatic tumors	Tumor not sensitive to light/photosensitizer, severe allergy to photosensitizer, contraindications to photosensitizer use, inaccessible tumor location for light delivery, severe coagulopathy	(84, 85)
HIFUA	Ultrasound	External ultrasonic waves converging at the focus to produce instantaneous high temperature	Non-invasive and without ionizing radiation, penetrating tissue to ablate deep tumors	High requirements for energy control, long time taken to ablate targeted lesions, risk of off-target in ablating irregular tumors, unsuitable for treating tumors in gas-containing organs	Applicable to non-invasive treatment of benign tumors, or as a palliative anti-tumor treatment, especially for patients with poor physical conditions	Ultrasound penetration issues (e.g., bone, lung, bowel gas), tumor location/size affecting focus, severe coagulopathy, poor systemic condition	(83, 86)
IRE	Short pulse electric field	High voltage pulse inducing apoptosis by forming irreversible holes in cell membrane	Destroying the cell membrane through pulsed electric field, not relying on thermal effects, less damage to blood vessels and nerves	Requiring special equipment and general anesthesia, complex operation, limited ablation range, potentially causing muscle contraction, expensive	Applicable to the tumor lesions adjacent to or around the key structures (blood vessels, nerve bundles, ureters, bile ducts), including pancreatic carcinoma, hepatic carcinoma, renal cell carcinoma, prostatic carcinoma	Proximity to heart (risk of cardiac arrest), severe coagulopathy, metal implants, severe systemic condition (e.g., cardiac arrhythmia)	(43, 81, 87)
CA	Very low temperature refrigerant	Formation of ice crystals through very low temperature, osmotic pressure damage	Easy to observe the ice ball boundary, larger and more precise zones of ablation, minimal effect on surrounding organs	Long ablation time, potential for bleeding after thawing, possible fatal complications such as severe hypothermia and cryoshock	Suitable for larger tumor lesions, or patients with coagulopathy situation	Proximity to vital organs/vessels, severe coagulopathy, severe heart and lung diseases, cold allergy or cold globulinemia	(81, 88, 89)

Preclinical studies have shown that in mCRC models, complete ablation of a single tumor lesion can exert an inhibitory effect on other nonablated lesions, exhibiting synergistic anti-tumor efficacy, particularly when combined with ICIs (60–64). However, in the clinical practice of treating mCRC with PA combined with ICIs, the reported focus remains predominantly on the ablation of LM (77). Therapeutic evidence for other sites (such as lung, peritoneum, adrenal gland, or bone metastases) remains limited or exploratory. This is primarily attributed to two factors: firstly, the liver is the most common site for metastasis of CRC; secondly, the percutaneous puncture and ablation of LM are relatively safer and technically simpler to perform. Moreover, in clinical application, the choice of PA modality is largely dictated by the anatomical location and lesion characteristics, irrespective of tumor type. This means that a given PA technique can effectively treat metastases from a range of tumors, and conversely, metastases from a single tumor type might be managed with multiple PA approaches. The selection of a specific PA method primarily depends on the size, number, and anatomical context of the target lesions, rather than their specific histology.

## 7 The optimal sequencing and timing of PA combined with ICIs

Determining the optimal sequence and timing for PA combined with ICIs is crucial for developing effective treatment strategies. First, most current studies report and recommend the sequential treatment strategy of PA followed by ICIs, aiming to enhance the sensitivity of systemic immunotherapy through local ablation and induce the abscopal effect (60–64, 67, 77). The potential benefit of PA followed by ICIs lies in ablation-induced release of TAAs and DAMPs, creating a highly immunogenic environment for subsequent ICIs application, thereby maximizing the abscopal effect of ablation. The main risk is the difficulty in timing of ICIs application: if the interval between ablation and ICIs use is too long, the peak of antigen exposure may pass; if too short, the optimal antigen presentation window might be missed. Second, certain researches suggest performing ICIs first, followed sequentially by PA, positioning ablation as a salvage or consolidative local treatment modality (59, 90). The potential benefit of performing ICIs before PA is the precise eradication of lesions that respond poorly to ICIs or have established local immune barriers. The main risk is that premature ICIs use might interfere with the establishment of the local immune response induced by ablation. Third, concurrent administration of PA and ICIs for the treatment of mCRC has also been reported (65, 66, 68). The potential benefit of concurrent use of PA and ICIs lies in the possibility of achieving the most rapid comprehensive anti-tumor effect, while the risk involves the superposition of local inflammation induced by ablation and adverse reactions caused by ICI therapy.

In summary, the approach of sequential ablation followed by ICIs application is currently the most commonly adopted regimen in PA combined with ICIs for the treatment of mCRC. However, several challenges remain, such as the frequency of ablation, the occurrence of incomplete ablation, and the optimal timing of sequential ICIs administration. Unfortunately, to knowledge, there has been no study designed to determine the frequency of ablation. As reported, for cases at risk of incomplete ablation, CCR2 antagonists may be considered to inhibit MDSCs recruitment (73). In multiple murine models of mCRC, ICIs (including antibodies targeting PD-1/PD-L1, TIGIT, and LAG-3) were all initially administered within 1 day after MWA (61–64). Another preclinical study exhibited that initial administration of anti-PD-1 antibody was within 1 day following RFA, MWA, CA or IRE (67). Therefore, in the treatment of mCRC with ICIs following PA, ICI therapy is often given within 1 day post-PA and the timing of ICI administration is independent of ablation modality and ICI class. Clinically, an important safety consideration for early ICI administration (within 1 day after PA) is the theoretical risk of exacerbated immune-related adverse events (irAEs) due to the acute inflammatory response induced by ablation. Vigilant monitoring for irAEs is crucial in patients receiving this early combination.

## 8 Clinical translation of PA combined with ICIs in the treatment of mCRC

### 8.1 pMMR/MSS subpopulation of mCRC

In mCRC, deficient mismatch repair (dMMR)/high microsatellite instability (MSI-H) accounts for approximately 4-5% (91–93). The efficacy of ICIs as monotherapy in dMMR/MSI-H mCRC has been supported by high-level evidences (94, 95). However, in clinical practice, the vast majority of mCRC patients exhibit proficient MMR (pMMR)/microsatellite stability (MSS) or low microsatellite instability (MSI-L), a population that often fails to benefit from ICI therapy (96). How to overcome the resistance of immunotherapy in mCRC patients with pMMR/MSS or MSI-L has become a research hotspot.

In a retrospective study (97), no objective response with the combination of regorafenib and anti-PD-1 antibody was found in unselected Chinese patients with pMMR/MSS mCRC, however, one patient who received RFA to treat liver and abdominal wall metastases before combination treatment ultimately experienced a significantly extended progression-free survival (PFS) of 9.2 months with stable disease (SD). The result of a single-arm phase II study showed that 41.7% of 12 patients with MSS mCRC who had undergone RFA plus dual ICIs achieved SD (77). Thus, PA combined with ICIs is potentially beneficial for the pMMR/MSS mCRC patients.

### 8.2 Low tissue TMB subpopulation of dMMR/MSI-H mCRC

Tumor mutational burden (TMB) serves as a critical biomarker associated with immunotherapy. High TMB (TMB-H) correlates with superior immune response, indicating that patients with TMB-H are more likely to benefit from ICI therapy. In unselected CRC populations, response to ICIs is associated with plasma TMB-H (98, 99) but not with tissue TMB-H (99–101). However, in MSI-H mCRC, tissue TMB is closely related to the objective response to immunotherapy, and patients with tissue TMB-H are more sensitive to ICIs therapy (102). Both univariate and multivariate analyses indicate that patients with tissue TMB-H experience a longer PFS after receiving ICI treatment (102). Therefore, PA combined with ICIs is potentially helpful for the low tissue TMB subpopulation of dMMR/MSI-H mCRC patients.

### 8.3 Wild-type POLE/POLD1 subpopulation of mCRC

POLE and POLD1 are the respective genes encoding DNA polymerase ϵ and δ, which are indispensable for maintaining the fidelity of DNA replication through their proofreading functions (103). Pathogenic POLE/POLD1 mutations lead to decreased fidelity of DNA replication, resulting in TMB-H or even ultra TMB-H (103, 104). Reportedly, the frequency of POLE/POLD1 mutations ranges from approximately 2.4% to 7.4% in overall CRC (104, 105) and 74% of tumors with POLE/POLD1 mutations are MSS or MSI-L (105). Furthermore, POLE/POLD1 mutations indicate a better response to ICI therapy and favorable prognosis in CRC patients (105, 106). Compared to dMMR/MSI-H mCRC patients treated with ICIs, those harboring POLE/POLD1 proofreading-deficient mutations exhibited a significantly higher overall response rate, as well as superior PFS and OS following ICI treatment (106). Consequently, the combination of PA with ICIs may be beneficial for the treatment of mCRC patients with wild-type POLE/POLD1.

### 8.4 RAS/BRAF-mutation subpopulation of dMMR/MSI-H mCRC

It was reported that 22% and 27% of dMMR CRCs harbored RAS mutation and BRAFV600E mutation respectively in a retrospective study (107). Within dMMR/MSI-H CRCs, the subsets harboring RAS or BRAF V600E mutations exhibit lower immunogenicity and an immune-depleted TME (107, 108), indicating that RAS/BRAF mutations predict a poorer immune response and reduced efficacy of ICIs. Accordingly, the approach of PA combined with ICIs is potentially advantageous for the RAS/BRAF-mutation subpopulation of dMMR/MSI-H mCRC patients.

### 8.5 CRC with LM

Recent studies suggest that, irrespective of the MMR/MSI status, CRCLM demonstrate inferior immune responses and treatment efficacy of ICIs compared to mCRC without LM (109–111). LM are characterized by: the propensity of Kupffer cells to polarize into tumor-promoting M2 phenotypes, increased secretion of the immunosuppressive cytokines interleukin-10 and transforming growth factor-β, the formation of an immune barrier constituted by the CXCL12/CXCR4 signaling axis and liver sinusoidal endothelial cells, enhanced expression of CD33, apoptosis of Fas^+^CD8^+^ T cells, reduced infiltration of CD8^+^ T cells, high levels of fibrinogen-like protein 1, and increased production of artemin (112, 113). The aforementioned characteristics of LM generate a unique immune-tolerant microenvironment, thereby leading to the relative insensitivity of CRCLM to ICI therapy. Similar to LM, primary hepatocellular carcinoma is characterized by poor immune cell infiltration and a strongly immunosuppressive TME, and most clinical trials using ICIs in combination with other therapies to treat hepatocellular carcinoma fail to meet their endpoints (114). The possible reasons are insufficient tumor immunogenicity, a deeply immunosuppressive TME, lack of robust T cell priming, and heterogeneity of patient response. Consequently, although PA combined with ICIs holds theoretical promise for CRCLM, a cautious approach is imperative in real-world application.

In summary, the aforementioned special subtypes of mCRC (pMMR/MSS, low tissue TMB, wild-type POLE/POLD1, etc.) are considered cold tumors and are not sensitive to ICI monotherapy. PA holds promise in converting these cold tumors into hot tumors, thereby enhancing sensitivity to ICI treatment. However, direct evidence demonstrating that these specific mCRC subpopulations can benefit from the combination therapy of PA with ICIs is currently lacking and awaits clarification through further clinical research.

## 9 Clinical studies investigating the combination of PA with ICIs for mCRC

Shi et al. (60) studied the tumor tissue samples of patients with CRCLM and found that RFA treatment for LM increased the infiltration of T cells and expression of PD-L1 in the primary tumor, which is consistent with the preclinical study findings described previously (60, 64). A retrospective study showed that one pMMR/MSS mCRC patient who received the treatment of RFA combined with anti-PD-1 antibody and regorafenib experienced a significantly extended PFS of 9.2 months (97).

In a multicenter, single-arm phase II study (77), 12 patients who had unresectable, liver-predominant mCRC with MSS after first- or second-line chemotherapy were treated with RFA. Only LM were ablated and the corresponding minimum ablated volume was 25 cm^3^, with a maximum of 120 cm^3^. The size of ablated lesions was between ≥3 and ≤6 cm in diameter. The number of lesions treated was left to the discretion of the investigator. Furthermore, the enrolled patients received immunotherapy of tremelimumab combined with durvalumab for 4 cycles, followed by durvalumab administeration alone every 4 weeks for a maximum of 8 months. As a result, no patients showed an objective response in any of the unablated metastases. The best overall response was SD in 5 (41.7%) patients and the median PFS (mPFS) was 2.2 months. The aforementioned study exhibited 0% objective response and a poor mPFS. The potential explanations lie in the inherently immunosuppressive TME of LM, an infrequent ablation schedule, and the fact that the majority of patients had undergone extensive prior systemic treatments. Although the results of above clinical research were not entirely encouraging, it established a crucial practical foundation for evaluating the clinical viability of RFA combined with ICIs in treating mCRC, particularly the subpopulation of MSS. Another clinical study suggested that HIFUA combined with ICIs for the treatment of CRCLM was feasible and safe in clinical practice, with manageable adverse events (78). However, the research included an insufficient number of cases, and further expansion of the sample size is required for a validation.

Unlike numerous preclinical studies, relevant clinical data about the combination of PA with ICI therapy for mCRC remain insufficient. Currently, multiple clinical trials are actively underway to provide evidence for the clinical application of PA combined with ICIs in mCRC treatment. **Table 2** summarizes the registration information of these trials.

**Table 2:** Ongoing clinical trials about the combination of PA with ICIs for mCRC.

Registration number	Phase	Ablation type	Combination interventions	Inclusion criteria	Sample size	Recruitment status	Estimated completion date	Primary/secondary endpoint	Registration number
ChiCTR2200066100	Phase 2	MWA	Camrelizumab; Cetuximab; Chemotherapy	CRC with liver/lung metastases, measurable metastatic lesions in addition to ablation lesions, wild-type RAS and BRAF gene, pMMR or MSS	43	Active, not recruiting	2025-10-24	Primary Outcome: the progression-free survival rate of 12 months Secondary Outcome: Objective response rate; Disease control rate; Progression-free survival; Overall survival; Safety; Efficacy predictive biomarker analysis; ctDNA dynamic monitoring	ChiCTR2200066100
ChiCTR2400082391	Phase 2	HIFUA	PD-1 inhibitor; Fruquintinib	Liver metastatic CRC progressing on prior at least second-line standard treatment, with at least one measurable metastatic lesion in addition to ablated lesions, pMMR or MSS	25	Recruiting	2026-06-30	Primary Outcome: Objective response rate Secondary Outcome: Safety; progression-free survival; Disease control rate; Overall survival	ChiCTR2400082391
ChiCTR2200058323	Phase 2	MWA	Tirelizumab; Fruquintinib	Metastatic CRC progressing on prior at least 2 lines of standard therapy, with suitable metastatic lesions for microwave ablation and at least one extracranial measurable lesion after ablation	25	Active, not recruiting	2024-09-30	Primary Outcome: Progression free survival Secondary Outcome: Objective remission rate; Disease control rate; Overall survival	
NCT04888806	Phase 2	MWA	Camrelizumab; Chemotherapy	Liver/lung metastatic CRC progressing after at least one systemic therapy, with measurable metastatic lesions after ablation, known KRAS, NRAS, BRAF and HER2 gene status	37	Active, not recruiting	2025-06-30	Primary Outcome:12-month progression-free survival Secondary Outcome: Objective response rate; Disease control rate; Progression-free survival; Overall survival; Safety	ChiCTR2100048492
NCT03101475	Phase 2	RFA	Tremelimumab;Durvalumab	Liver metastatic CRC with liver metastases amenable to ablation, measurable metastatic lesions after ablation, and availability of tumor sample for biomarkers testing (MSI, PD-L1, etc)	22	Completed	2022-02-23	Primary Outcome: Best overall immune response rate (iBOR) of lesions not treated by ablation/radiotherapy including the extrahepatic lesions according to iRECIST (with response confirmation) Secondary Outcome: Best overall immune response rate of liver lesions not treated with local therapy according to iRECIST (with response confirmation); Safety; Stable disease duration; Progression free survival; Best overall response rate of lesions not treated by ablation/radiotherapy including or not the extrahepatic lesions according to RECIST v1.1(with response confirmation); Response duration; Overall survival	NCT03101475
NCT04888806	Phase 2	MWA	Camrelizumab; Chemotherapy	Liver/lung metastatic CRC progressing after at least one systemic therapy, with measurable metastatic lesions after ablation, known KRAS, NRAS, BRAF and HER2 gene status	37	Not yet recruiting	2025-06-30	Primary Outcome:12-month progression-free survival Secondary Outcome: Disease control rate; Progression-free survival; Objective response rate; Overall survival	NCT04888806
NCT05057052	Phase 2	CA	Sintilimab; Regorafenib	Liver metastatic CRC progressing on prior at least two systemic therapies, with at least one measurable lesion after ablation	25	Recruiting	2026-09-25	Primary Outcome: Objective response rate Secondary Outcome: Overall survival; Progression-free survival; Adverse Events; Disease control rate; Duration of response	NCT05057052
NCT05485909	Phase 2	RFA	Toripalimab; Regorafenib	Liver metastatic CRC with pMMR or MSS/MSI-L after previous systemic treatment that must contain fluorouracil, oxaliplatin and irinotecan, with or without targeted therapy, in addition to the ablated lesion and the measurable lesion outside the liver, there is at least one measurable lesion in the liver	32	Recruiting	2024-06-30	Primary Outcome: Objective Response Rate Secondary Outcome: Progression free survival; Overall survival; Disease control rate; Duration of response; Time to disease progression	NCT05485909
NCT04116320	Phase 1	HIFUA	PD-1 inhibitor	Metastatic CRC having failed or having contraindication to standard therapies, with measurable disease and suitable lesions for ablation	5	Terminated	2023-08-15	Primary Outcome: To assess the safety and toxicity of focused ultrasound ablation administered alone or in combination with PD-1 antibody blockade; To estimate the proportion of patients with increased CD8^+^T cell infiltration of spot focused ultrasound ablation-treated metastasis Secondary Outcome: To estimate the proportion of patients with increased CD8^+^T cell infiltration, after spot focused ultrasound ablation, in untreated metastasis, when available	NCT04116320
NCT06590259	Phase 2	Multimodal thermal ablation	Sintilimab; Chemotherapy; Bevacizumab or Cetuximab	Liver metastatic CRC progressing after first-line treatment with liver lesions suitable for ablation	20	Recruiting	2027-12	Primary Outcome: Progression free survival Secondary Outcome: Immune indicator analysis; Overall survival; Local progression free survival; Objective response rate; Rate of adverse events	NCT06590259
NCT04202978	Phase 1/2	RFA	Camrelizumab; Apatinib; Chemotherapy	Metastatic CRC with 3 or more metastases only in the liver which are confirmed to be unresectable and evaluable or measurable	23	Recruiting	2021-11-01	Primary Outcome: R0 resection rate Secondary Outcome: Objective response rate; Pathologic complete response	NCT04202978
NCT02437071	Phase 2	RFA	Pembrolizumab	Metastatic or recurrent CRC after prior two or more standard available therapies, with at least one tumor suitable for ablation and at least one measurable lesion	34	Completed	2024-09-20	Primary Outcome: Response rate Secondary Outcome: Toxicity	NCT02437071
NCT03122509	Phase 2	Ablation	Tremelimumab;Durvalumab	Metastatic CRC progressing on prior at least two standard chemotherapy regimens, with at least one tumor suitable for ablation and at least one measurable lesion	25	Completed	2021-04-28	Primary Outcome: Overall response rate	NCT03122509
NCT06889610	Phase 2	Multimodal thermal ablation	Cadonilimab; Fruquintinib	Liver metastatic CRC progressing on prior standard second-line drug therapy, with at least one measurable lesion in addition to the ablation lesions	95	Recruiting	2028-12-30	Primary Outcome: Objective response rate Secondary Outcome: Overall survival; Disease control rate; Duration of response; Safety and tolerability; Progression free survival	
NCT06630624	Phase 1/2	MWA or IRE	IP-001	Progressive or stable liver metastatic CRC after at least 1 line standard systemic treatment	120	Recruiting	2031-08-01	Primary Outcome: Disease control rate; Maximum tolerated dose; 1-year distant progression free survival Secondary Outcome: Duration of best overall response; Distant progression free survival; Overall survival; Progression free survival	

## 10 Discussion

PA technology, as a localized therapeutic approach, demonstrates unique advantages in the treatment of mCRC. It not only ablates local lesions and reduces tumor burden, but also activates T-cell anti-tumor immune response by inducing the release of TAAs and DAMPs from ablated lesions (6–11, 115). Furthermore, PA possesses the potential to sensitize CRC to ICIs through robust immunomodulatory effects. On the one hand, PA promotes the migration of effector CD8^+^ T cells to the nonablated tumor regions, thereby increasing the number of cytotoxic T lymphocytes in the TME and simultaneously ameliorating local tumor immunosuppression (10, 11, 114). On the other hand, PA also upregulates the expression of immune checkpoints such as PD-L1, PD-1, LAG-3 and TIGIT in the TME of distant untreated tumor lesions (60–64). The combination of PA and ICIs can significantly improve the immunosuppressive microenvironment of the tumors, enhance the immune activity of T cells, and thereby more effectively eliminate residual tumor cells and inhibit the growth of nonablated lesions.

Currently, PA combined with ICIs for the treatment of mCRC is still in the exploratory phase, and requires further in-depth research and refinement. The clinical application of this combined approach in the treatment of mCRC also requires the accumulation of more clinical research data. Future research should focus on the following aspects. (1) In-depth mechanistic studies: Although numerous animal studies have demonstrated that PA combined with ICIs exerts synergistic anti-tumor effects by reshaping an immunostimulatory TME, the underlying mechanisms have primarily been elucidated through flow cytometry and single-cell sequencing (59–68, 70–72). Therefore, the specific molecular mechanisms require further validation through in-depth molecular biology research. It is essential to further elucidate the underlying mechanisms of PA combined with ICIs, and to explore strategies for more effectively and efficiently mobilizing the host’s anti-tumor immune response. (2) Identifying optimal patient populations: In clinical practice, not all patients with mCRC derive benefit from the combination of PA and ICIs. Consequently, there is an urgent need to leverage biomarkers—such as TME characteristics, immune cell infiltration profiles, immune-related biomarkers, and mutational status—to identify patient subgroups most likely to respond to this combination therapy. (3) Exploring the optimal sequencing and timing of PA plus ICIs and developing individualized therapeutic strategies: Although most studies indicated that in the treatment of mCRC with PA combined with ICIs, PA was administered sequentially before ICIs (60–64, 67, 77), and ICIs were generally given within 1 day after PA (61–64, 67), the optimal sequencing and timing for the combination of PA and ICIs have yet to be fully elucidated. In addition, it is essential to integrate the patient-specific tumor characteristics and immune status to explore the optimal PA method and timing of ICIs administration, thereby maximizing the activation of anti-tumor immune responses and achieving precision medicine. (4) Multicenter randomized controlled clinical trials: Compared with preclinical findings, clinical data regarding the combination of PA and ICIs for mCRC are currently significantly limited; existing clinical trials are primarily single-arm, phase 1–2 studies. Therefore, it is imperative to conduct multicenter, large-scale, randomized controlled phase 3 trials to provide robust evidence-based medical support for PA plus ICI therapy in mCRC. (5) Nanomaterials for enhanced antigen presentation: Future strategies should utilize nanomaterials to boost antigen presentation post-ablation, improving antigen-presenting cell (APC) activation and T cell responses, particularly for mCRC with immune evasion. (6) Radiomics to predict post-ablation immune response: Radiomics offers a non-invasive means to predict patient response to the therapy of PA + ICIs by analyzing imaging features, enabling personalized treatment selection for mCRC.

We believe that advancements in research and technology will further solidify the potential of PA combined with ICIs as a novel therapeutic strategy for mCRC, offering enhanced survival benefits to an expanded patient population.

## 11 Conclusion

PA is a locoregional anti-tumor treatment approach that, while eliminating target lesions, can activate anti-tumor immune responses of CD8^+^ T cells and upregulate the expression of immune checkpoints in the TME of nonablated tumors. Preclinical studies have shown that in mCRC, PA in combination with ICIs can produce synergistic anti-tumor effects, providing a theoretical basis and experimental evidence for the clinical application of this combination therapy. For the clinical translation of PA combined with ICIs, immediate launch of large-scale multicenter studies is essential to evaluate the safety, efficacy, optimal sequencing and timing, and to specify the patient subgroups achieving maximal clinical benefit.
